# Sex and Character

**Published:** 1906-12

**Authors:** 


**Sex and Character.** By OTTO WEININGER. Authorised translation from the Sixth German Edition. Pp. xxii., 356. London: Wm. Heinemann. 1906.

The study of the author and of his work gives results of equal interest. Weininger appears to have suffered from one of the various forms of *degénéré supérieur*, first fully described by French alienists. Here was a youth brilliant in parts, certainly without recognisable insanity, but with irregular limitations of mental horizon, who was possessed by overmastering convictions on certain subjects, which he allowed to pervert his premises and coerce his logical processes. He has taken heavy toll from many writers where their theories have accorded with his own, and has perhaps not invariably acknowledged them sufficiently, but he has so thoroughly assimilated their work and woven it so deftly into the texture of his own that his book shows in no place the evidence of a compilation. Although the work is for the most part deficient in physical basis, and many of his theories are crude and lacking sufficient support, although the whole credence he gives to many of his quoted authorities is doubtfully justifiable, and hysterical the insistence on many of the inferences he draws, yet there is nevertheless an abundance of shrewd, even brilliant observation, which often convinces in spite of the unpalatable truths it discloses. The author's untimely end by suicide, the evidence of an enormous digestion of general and special psychological literature in proportion to his youthful age, the keen and unremitting virulence of his attack on woman, the obvious avowal of a defensive attitude, if not a partisanship, with regard to certain forms of sexual perversion, have secured for this book an amount of attention which we think may have been to some extent too generously given.

Apart, however, from the pursuance of his main theory, there are sections of more general interest on which eulogy has been well bestowed. Such are the chapters on Logic and Ethics, Memory, Talent, and Genius, the "Ego" problem, which are at least worthy the most careful study. These reveal an occasional transcendental penetration and lucidity of expression quite remarkable. It appears to us that there has been at work in the author's mind a latent hostility to womankind, so unsleeping, so unsparing, that it needs must have proceeded from an unavowable, if recognised, sense of injury suffered in relationship to this sex. Weininger's bitterness is that of the renegade or of one who stigmatises in others the hidden and deplorable vices or defects of his own nature. In the author's own words there is some suggestion of this attitude. Thus he writes: "We hate only qualities to which we approximate, but which we realise first in other persons." Again: "Those men who claim to understand women are themselves very nearly women. . . . We have to rely upon the female element in man; the principle of sexually intermediate forms is the authority for what we know about woman through man." There are several passages in the book from which inference may be drawn that one of the curious anomalies of sex, mental, physical, or both, of which the author treats, may have been his unfortunate and embittering lot. We can here only give the briefest mention of his theories. Weininger assumes that perfect male and perfect female are ideal conceptions; each sex has a varying proportion of its opposite merged with it. That is, all actual organisms are both homo and hetero-sexual, and homo-sexuality is merely the sexual condition of those intermediate forms that stretch from one ideally sexual condition to the other. Not only coarse anatomical signs may reveal these intermediary states, but it is asserted that every cell in the body is sexually characteristic and may exist in all conditions from complete masculinity to complete femininity. Thus Weininger assumes that the (practically) complete male will have some female elements, but the larger proportion of masculine; the woman the reverse. True sexual union must imply that male and female elements must be in complementary proportion to each other; the larger the proportion of maleness in a man, the larger should be that of femininity in the female. Varying in relation as these complementary parts may be, the author insists that man may be almost completely psychically female, but woman remains fundamentally feminine, and her masculinity can never attain to a corresponding extent; she has masculine traits, that is all.

But it is to this relative maleness in women that Weininger refers any intellectual qualities they may possess. All women, he writes, who are truly famous and of conspicuous mental ability at the first glance reveal some of the anatomical characters of the male, have almost invariably been what he describes as sexually intermediate forms. This doctrine is the basis of most of his arguments throughout the book. Having established man, though admittedly not perfect, as the moral and intellectual being, Weininger proceeds by every legitimate and illegitimate use of logic to descry woman as a non-moral, non-intellectual being, having no meaning apart from the sexuality of man. "Her existence is bound up with the Phallus, her welcome master and supreme lord," he boldly says. So, according to him, woman is shameless, organically untruthful ("it is quite wrong to say that women lie, that would imply that they sometimes speak the truth"), without sympathy, without soul, without continuity in memory, unable to think but in unclarified ideas ("henids"), without principles, logic, or judgment. She is devoid of personality, freedom, character, or will; is too low in the moral scale to be designated criminal, and has only one positive general characteristic, that of matchmaking, or the furthering of sexual union. Above all, woman is sexual, and nothing but sexual throughout. Being such, she cannot recognise her sexuality, and attempts to deny it. From these assumptions the author derives his theory of hysteria. The basis of this is the passive and entire acquiescence of woman in the masculine and wholly conventional estimate of herself, instead of allowing her own proper character free play. Hysteria is the bankruptcy of this superficial sham self, which has been only assumed; it is the organic crisis of the organic untruthfulness of woman. It is psychological sexual traumatism. Weininger makes very plain his views on sexual abstinence; that the world's human population should come to a close is preferable to the moral murder of man and woman by sexual congress. Marriage to him, in its existing form, is as incompatible as free love with the highest interpretation of the moral law. Motherhood is no sacred ideal for him; the prostitute as a phenomenon, not as a person, is much more estimable. To him every form of fecundity appears offensive, and he denies that any person can honestly convince himself that there is any need to provide for the continuity of the race. Women too must spontaneously relinquish sexual relationships with men; thus woman as woman must disappear, "and until that has come to pass there is no possibility of establishing the Kingdom of God upon earth." After having ruthlessly stripped woman of every vestige of valuable or estimable characteristics, the author absolves her from the opprobrium of any active scelerity by recognising that her guilt has been made by man, and to him is the blame. Weininger considers his aim in writing this book to be the emancipation of woman from her vile and voluntary serfdom, and thus to constitute the highest honour paid to the sex. Women, considering his views as to their sexual abstinence, may perhaps regard his tribute in the melancholy light of an obituary monument to their sex.

We must, however, give warning that the extreme views crudely presented in this place must not be allowed to stigmatise the work as a whole. Impetuous, biased, even dominated by some conviction to which we have no certain clue as is Weininger's book, yet there is a fitful gleam of genius illuminating its pages here and there, an unsparing effort to attain full consciousness and comprehension of his subject, and an unshrinking responsibility for his terrible indictments. The evidence of his highest thought is contained in the chapters before specially mentioned; his faults are those of a morbid egoism and a jaundiced attitude of mind, perhaps the result of a baffled eroticism, which has led him to the pursuance of a theory with fanatical extravagance.

In conclusion, we must repeat the caution given by the author in his preface, that the book is of a quality not to be understood at the first glance. The production of the book is all that could be desired, and the translator's work performed with masterly skill.

